# Longitudinal pattern of multimorbidity in older adult population: latent transition analysis in 34 countries

**DOI:** 10.1093/aje/kwaf129

**Published:** 2025-06-13

**Authors:** Ridho Al Izzati, Eduwin Pakpahan

**Affiliations:** Applied Statistics Research Group, School of Engineering, Physics and Mathematics, Northumbria University, Newcastle upon Tyne NE1 8ST, United Kingdom; Applied Statistics Research Group, School of Engineering, Physics and Mathematics, Northumbria University, Newcastle upon Tyne NE1 8ST, United Kingdom

**Keywords:** multimorbidity, latent transition analysis, cross-country comparisons, older adults

## Abstract

Multimorbidity has become a global public health concern, yet cross-national comparisons remain limited, especially in longitudinal settings. This study investigates the longitudinal patterns and transitions of multimorbidity status of people over age 50 in 34 countries. Utilizing comparable health indicators across countries, we examine chronic health conditions (hypertension and diabetes), cognitive function, physical ability, and self-report of general health. Using latent transition analysis, we identify a pattern of multimorbidity and classify it into three classes: mild, moderate, and severe multimorbidity. Mild multimorbidity is characterized by a lower prevalence of 3 morbidities out of 5, while severe multimorbidity is characterized by a higher prevalence across all health conditions. Moderate multimorbidity falls between these 2 extremes. Our findings reveal substantial variation in these classes across countries, with diabetes and hypertension emerging as the predominant condition among older adults with severe and moderate multimorbidity, respectively. Over time, both severe and moderate multimorbidity tend to increase, with similar transition probabilities from mild to more severe categories across countries. Covariate analysis indicates that men and low-educated individuals are more likely to experience severe multimorbidity. These results underscore the importance of understanding multimorbidity patterns and dynamics for effective public health planning and healthcare services.

This article is part of a Special Collection on Cross-National Gerontology.

## Introduction

Multimorbidity is characterized by the coexistence of 2 or more chronic diseases in an individual and is increasingly prevalent among the older adult population.^1^ Multimorbidity has become a public concern, because it affects overall quality of life,^2^ such as increased mortality^3^ and increased healthcare utilization and expenses.^4^^,^^5^

While the prevalence of multimorbidity increases with age, its variation appears to differ widely across countries (or continents). For instance, in high-income countries, the average prevalence of multimorbidity among adults is about 39% and 43% in Europe and North America, respectively.^6^ The average prevalence of multimorbidity among the older adults is much higher in that region about 53%-66%, respectively.^7^ Meanwhile, the average prevalence of multimorbidity among adults in lower-middle-income and upper-middle-income countries is about 32%-38%, respectively. The average prevalence of multimorbidity among the older adults in the same regions is about 48%-46%, respectively.

Most studies focused on the prevalence of multimorbidity using populations in specific countries or cross-sectional data.^8^ They have different sets of morbidity indicators and target populations that impede the direct comparison between countries. To the best of our knowledge, comparable cross-country evidence remains limited, especially in longitudinal settings and fair comparison between high-, upper-middle, and lower-middle-income countries. This study aims to identify the longitudinal pattern of multimorbidity and its transition over time in 34 countries. We use a model-based approach called latent transition analysis (LTA) to estimate the latent class of multimorbidity among the older adult population and its transition for three periods of time that span an average of 5 years.

We hypothesize that the co-occurrence of morbidity among populations can have a latent (or unobserved) group that can give us a different insight or perspective regarding multimorbidity. For example, multimorbidity can be dominated or driven by certain health conditions, or multimorbidity can have a different intensity among populations (ie, low vs high prevalence of two chronic diseases). Understanding the types and trends of these morbidities across time is essential as life expectancy continues to rise globally. This underscores the importance of comprehending the patterns and transitions of multimorbidity for effective public health planning and healthcare service provision.

This study contributes to literature in several ways. First, we use various dimensions of health including chronic diseases, cognitive function, physical ability, and self-assessment of health. Second, it uses not only objective measures but also a subjective measure of health as a factor of multimorbidity. Third, this study coverage encompasses various socio-economic and healthcare contexts across the nation not only in high-income countries but also lower-middle-income countries. Lastly, this study provides insights into the dynamics (trend and transition) of latent multimorbidity patterns over time using a large set of longitudinal surveys.

## Methods

### Data

One challenge in cross-country analysis is the fair comparability of the health indicator that captures various dimensions of health. Comparability is not only across countries but also across different times. In this study, we utilize a comparable health indicator across countries that captures the aspects of cardiovascular (represented by hypertension), metabolic and endocrine (represented by diabetes), musculoskeletal (represented by physical difficulty), and mental health (represented by cognitive impairment). Those aspects are the most common health conditions that are being used to assess morbidity in more than 80% of 452 studies regarding multimorbidity.^9^ To capture other health conditions, we also use a comparable self-rated health report question, that is, one of the important health outcomes that can capture the general health state of an individual.^10^ Each health indicator correlates with each other indicating the interrelationship between one morbidity and another morbidity and its possible relationship to drive multimorbidity conditions. For example, individuals with diabetes tend to have cognitive impairment^11^ as well as physical ability.^12^

We use Health and Retirement Studies (HRS) for the US,^13^ English Longitudinal Study of Ageing (ELSA) for England,^14^ Survey of Health, Ageing, and Retirement in Europe Life History (SHARE) for 28 countries in Europe,^15^ Indonesian Family Life Survey (IFLS) for Indonesia,^16^ China Health and Retirement Longitudinal Study (CHARLS) for China,^17^ Mexican Health and Aging Study (MHAS) for Mexico,^18^ and Costa Rican Longevity and Healthy Aging Study (CRELES) for Costa Rica (Except for HRS and IFLS, all datasets used are harmonized datasets provided by the Gateway to Global Aging Data (www.g2aging.org) team (all data versions are listed in the Supplementary Materials Appendix S1).^19^ All datasets are designed to be comparable with the HRS by using similar questionnaires to assess the health outcomes used in this study. We performed pre-statistical harmonization following the procedures outlined in the Gateway to Global Aging Data user guide (https://g2aging.org/user-guide).^20-23^ Each dataset employs a multi-stage probability sampling design, incorporating geographical stratification and demographic groups. To account for variations in survey design and to ensure the sample’s representativeness, we apply the design weights provided by the dataset.

### Inclusion criteria of the sample

We employ the following inclusion criteria for the sample. First, we define the older adults as individuals who are 50 and above and have no missing values of all variables during the baseline period. Second, our sample is present at least 2 times in our three points of observation. The inclusion of individuals aged 50 and above as older adults is based on 2 reasons. First, it aligns with the eligibility criteria commonly used in many aging-focused studies, particularly those utilizing the same datasets.^24^^,^^25^ By adopting this definition, our study captures the early effects of aging while ensuring comparability with similar studies in the field. Additionally, age 50 marks a critical transition period in life, during which many chronic health conditions and the early stages of multimorbidity begin to emerge.^26-28^ This approach allows us to better examine the progression of health outcomes as individuals age. We used the most recent 3 periods of each dataset whenever possible. Except for Indonesia, all countries have 3 periods of observation (The survey year for all countries in Europe is from 2017 to 2022, from 2014 to 2019 for England, from 2012 to 2016 for the United States, from 2013 to 2018 for China, from 2005 to 2009 for Costa Rica, and from 2012 to 2018 for Mexico (see Table S1 in Supplementary Materials for the complete list). Meanwhile, the dataset for Indonesia is only available for the last 2 survey rounds. The survey period for Indonesia is in 2007 and 2014. The gap between the survey for Indonesia is 7 years which is the longest gap in our study case. Therefore, we consider the latest survey period for Indonesia as the second follow-up time point, aligning it with the second follow-up in other datasets in this study, rather than the first follow-up.). On average, the gap between the baseline and the second follow-up surveys for all countries is 5 years. From the final sample, there is an average of 75% up to 80% individuals present in all three-time points for each dataset, while the rest are present at least two times. This inclusion criterion optimizes the sample size by allowing the use of partially complete cases, rather than limiting the analysis to only fully complete cases. We use the full information maximum likelihood (FIML) estimator to handle missing data. While attrition may appear to be a significant issue, several studies that utilize the same dataset indicate that non-random panel attrition is not a major concern.^29^ Notably, the estimation results are unaffected by potential attrition problems, even in long-term analysis of health shocks.^30^

### Health outcomes

We use 5 binary variables indicating morbidity: diabetes, hypertension, cognitive impairment, physical difficulty, and low self-rated health. The variable takes a value of 1 if an individual has morbidity, while 0 indicates the absence of morbidity. Diabetes and hypertension are chronic diseases defined by a self-report, that is, based on a doctor’s diagnosis. Cognitive impairment is defined by word recall test scores below 5 (out of 10) for immediate word recall and below 4 (out of 10) for delayed word recall.^31^^,^^32^ Physical difficulty is defined as an individual having any difficulties in a set of activities of daily living (ADL) questions (We include 12 ADL questions that capture activities of daily living, instrumental activities of daily living, and other functional limitations.). Lastly, low self-rated health is defined as very poor, poor, or fair health status from self-report of general health questions (In the harmonized dataset, the options for self-reporting of general health status are excellent, very good, good, fair, and poor. We use the same variable and definition for all datasets except for CHARLS and IFLS. For CHARLS, we define low self-rated health using options very poor and poor categories, while for IFLS, we define low self-rated health using options very unhealthy and somewhat unhealthy.).

We also use demographic variables: gender, age, and education as covariates in the estimation. For education, we define a binary variable high education with a value of 1 for the individual who has at least secondary level while 0 for the individual who has less than secondary education (See Table S2 in the Supplementary Materials for the prevalence of health outcomes, mean of demograpic variables, and sample size).

### Statistical analysis

**Figure 1:**
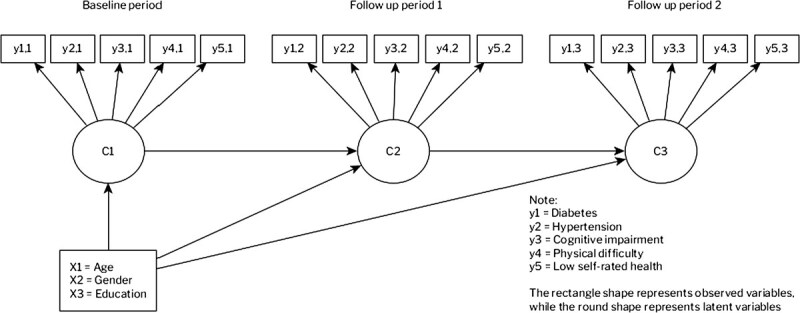
LTA model.

First, our analysis is based on descriptive statistics of multimorbidity from raw data. Second, we conduct a LTA to estimate the latent group of multimorbidity within our sample. Categorical latent classes are fitted at once using data from three time points as shown in Figure 1.^33^ Latent transition analysis is a longitudinal extension of latent class analysis, a commonly used analysis to identify multimorbidity patterns among populations.^34-38^ As a model-based approach, LTA results in more parsimonious and more interpretable findings when identifying unobserved patterns in multimorbidity. Latent transition analysis also allows for examining the transitions between different health states (latent classes) over time, providing insights into the dynamic nature of health and disease progression in older adults.

In our study, there are 32 768 (2^5×3^) combinations of responses to 5 health outcomes with 3 time points. The following model 1 expresses how the probability of observing a particular vector of responses ($P\left(Y=y\right)$) is represented.^39^ The first part is ${\delta}_{s_t}$ which represents the prevalence of latent status $s$ at time $t$, where $S$ is the total number of classes estimated and $T$ is the number of time points. The second part is ${\tau}_{s_T\mid{s}_{T-1}}$ which represents the probabilities of transitioning to a latent status at a particular time conditional on latent status membership at the previous time. ${\delta}_{s_t}$ and ${\tau}_{s_T\mid{s}_{T-1}}$ are the main parameters of interest in our study. The third part of the model is ${\rho}_{j,{r}_{j,t}\mid{S}_t}$ which represents the probability of response ${r}_{j,t}$ (1 = yes and 0 = no for each health outcome) to observed variable $j$, conditional on membership in latent status $s$ at time $t$. In our case, $j$ is observed health outcomes with total $J$ (five) variables.


(1) \begin{equation*} P\left(Y=y\right)=\sum_{s_1=1}^S\dots \sum_{s_T=1}^S{\delta}_{s_1}{\tau}_{s_2\mid{s}_1}\dots{\tau}_{s_T\mid{s}_{T-1}}\prod_{t=1}^T\prod_{j=1}^J\prod_{r_{j,t}=1}^{R_j}{\rho}_{j,{r}_{j,t}\mid{S}_t}\ . \end{equation*}

We estimate model 1 using FIML estimator. Full information maximum likelihood utilizes all available information in the dataset without discarding any missing values. This approach provides unbiased estimates and is more efficient than alternative methods (such as listwise or pairwise deletion).^40^ We use an iterative estimation with 500 random sets of starting values to avoid local maxima.^41^ We also constrain the probability across time to allow a straightforward interpretation of the transition probabilities across the 3 time periods (measurement invariance). Besides class interpretation, the restriction on the item probability helps with model identification and ensures the latent class has the same meaning over time. Bayesian information criterion (BIC) and entropy of the latent class model are used as an indicator of model fit.^42^ Meanwhile, to determine the number of classes to use in the analysis, we use 3 assessments, including the information criterion (BIC), item probability, and interpretability of the class.

We also include covariates in the estimation. For cross-country comparison, the country is divided into groups based on income and geographical regions. The countries are categorized into high-income (HIC), upper-middle-income (UMIC), and lower-middle-income (LMIC) based on the World Bank income classification.^43^ Meanwhile, the United Nations Standard Country or Area Codes are used to separate countries into geographical regions (Our dataset includes 28 high-income, 5 upper-middle-income, and 1 lower-middle-income countries. We have also identified seven regions: Western Europe, Eastern Europe, Northern Europe, Southern Europe, North America, Central America, and Asia. We include Cyprus and Israel in the Southern Europe category for the analysis.).^44^ We use Stata version 17 for data preparation and descriptive analysis and Mplus version 8.11 to run the latent class and probability transition model.

### Exploratory analysis of latent class of multimorbidity

Estimating LTA for a complex model with a large sample and three periods is computationally intensive due to its iterative nature. Therefore, we conducted an exploratory analysis of latent classes and transitions using only 2 time points. The model is estimated with 2-7 classes, and model fit is assessed to identify the optimal number of classes.

Before we fit the latent transition model for three periods, we show the exploratory results of a latent class using 2-time points. First, we use information criterion (BIC) from the model estimation which is shown in Figure S1 in the Supplementary Materials. The BIC has decreased substantially from a 2-class model to a 3-class model for all samples as well as for each country model. The BIC continues to decrease up to a 6-class model and then increases in a 7-class model. However, the decrease in BIC is much larger from 2- to 3-class models compared to the decrease from other class models.

Second, we use item probability to assess the class. For this purpose, we use the estimation of the HRS dataset as an illustration (see Figure S2 in Supplementary Materials). The item probability of the 2-class model shows parallel patterns with different prevalence levels for almost all morbidity indicators. These patterns can be categorized into low- and high-level groups with 2 unique patterns: hypertension for the high-level group and physical difficulty for the low-level group. Moving to the 3-class model adds another parallel trend for at least 4 indicators. The new pattern introduces a new level with a unique pattern, such as diabetes being the leading indicator in this case. Although the BIC continues to decrease for larger classes, the improvement in the parallel line of item probability is not substantial beyond 3 classes, resulting in a duplicate pattern of leading indicators. The third reason relates to the interpretability of the classes. Upon examination, the 3-class model showed a meaningful interpretation due to the substantive meaning of the classes. Assessing the transition probability for the 3 classes in LTA is also straightforward. Lastly, because the item probability shows classes with high and low levels of prevalence, we can classify the 3 latent classes as mild, moderate, and severe multimorbidity.

## Results

### Descriptive statistics of health conditions

**Figure 2:**
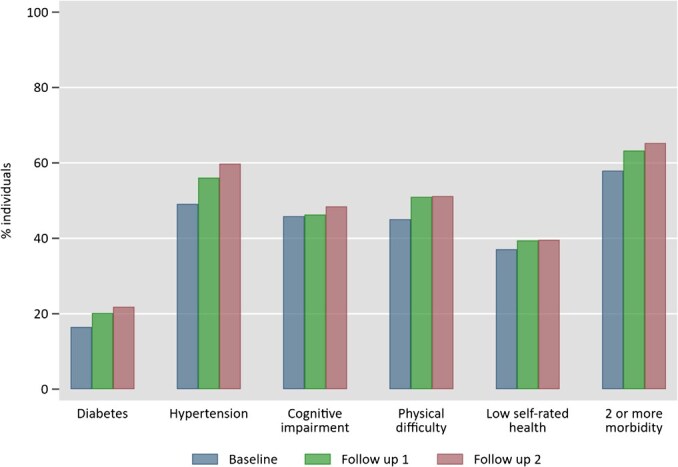
Prevalence of health conditions combining 34 countries (%).

In Figure 2, we present the raw tabulation of health outcomes or morbidities for all countries used in this study. Almost all morbidities have increased in prevalence from the baseline to follow-up periods. The health condition with the highest prevalence (up to 60%) and growth rate (12 percentage points) is hypertension. In contrast, diabetes has the lowest prevalence (around 20%) compared to the other 4 outcomes; however, it has also experienced a higher rate of increase over time. Almost half of older adults in our sample have a cognitive impairment with a slightly increasing rate over time. Half of the respondents reported physical difficulty with a higher increase from baseline to the second follow-up period. Around 40% of the samples report a poor general health status which tends to increase over time.

In Figure 2, it is evident that the prevalence of older adults with 2 or more morbidity has increased over time from 57.9% at the baseline to 65.4% at the second follow-up period. However, this tabulation alone does not provide insight into potential latent subgroups that may be present among the older adults in our sample. The next section will outline the findings of the analysis of latent groups.

### Latent class of multimorbidity

Next, we then estimate the latent transition model for 3-time points using 3 latent classes. We estimate the model for each country and for the pool dataset. For the pool dataset, we pool all countries’ data together and estimate the model by controlling for country dummies, with Germany as the base, to account for country heterogeneity. Table 1 shows the conditional probability of each health status within the latent class for the pool dataset. The mild multimorbidity class is characterized as a group of the older adults that has a relatively lower prevalence of only 3 morbidities. The dominant health problem among this group is cognitive impairment. Moderate multimorbidity is indicated by the group of older adults with a slightly higher prevalence of 4 morbidities compared to the mild category. This moderate category is dominated by the group of older adults with hypertension conditions with a prevalence of 100%. Meanwhile, severe multimorbidity is indicated by the group of older adults with a relatively higher prevalence on all health statuses. This severe category is dominated by the group of older adults with diabetes and hypertension with a prevalence of 100% and 79%, respectively.

**Table 1:** Item probability combining 34 countries.

**Latent class**	**Health status**				
	**Diabetes**	**Hypertension**	**Cognitive impairment**	**Physical difficulty**	**Low self-rated health**
Mild	0.01	0.00	0.43	0.37	0.26
Moderate	0.00	1.00	0.49	0.54	0.43
Severe	1.00	0.79	0.52	0.64	0.57

The item probability pattern of each country is largely similar to the pool dataset (refer to Figure S3 in the Supplementary Materials). Table 2 shows the prevalent health conditions within a category for each country. Similar to all-country patterns, diabetes is the most common health condition among the group with severe multimorbidity in 20 countries (59% of total countries), hypertension is the most common health condition among the group with moderate multimorbidity (82% of countries), and cognitive impairment is the most common among the group with mild multimorbidity group (62% of total countries). In some other countries, hypertension and physical difficulty are the most common health status in the older adults with severe multimorbidity group. However, different patterns also emerge in other countries. For example, in countries like Lithuania and Latvia, low self-rated health assessment is the dominant health concern for this group. Meanwhile, Indonesia stands out as the only country where cognitive impairment is the main health condition for those with moderate health issues. The United States, England, and many Western European countries have physical difficulty as the dominant health condition for people with mild multimorbidity.

**Table 2:** Dominant health conditions within the category for each country.

**Country**	**Latent class of multimorbidity**		
	**Mild**	**Moderate**	**Severe**
England	Physical difficulty	Hypertension	Diabetes
Austria	Physical difficulty	Hypertension	Hypertension
Germany	Physical difficulty	Hypertension	Diabetes
Sweden	Cognitive impairment	Hypertension	Diabetes
Netherlands	Cognitive impairment	Hypertension	Diabetes
Spain	Cognitive impairment	Hypertension	Diabetes
Italy	Cognitive impairment	Hypertension	Hypertension
France	Physical difficulty	Hypertension	Diabetes
Denmark	Cognitive impairment	Hypertension	Physical difficulty
Greece	Cognitive impairment	Hypertension	Physical difficulty
Switzerland	Cognitive impairment	Hypertension	Diabetes
Belgium	Physical difficulty	Hypertension	Physical difficulty
Israel	Cognitive impairment	Hypertension	Hypertension
Czech Republic	Physical difficulty	Hypertension	Diabetes
Poland	Cognitive impairment	Hypertension	Diabetes
Luxembourg	Physical difficulty	Hypertension	Diabetes
Hungary	Hypertension	Diabetes	Physical difficulty
Portugal	Cognitive impairment	Hypertension	Diabetes
Slovenia	Cognitive impairment	Hypertension	Diabetes
Estonia	Low self-rated health	Hypertension	Diabetes
Croatia	Cognitive impairment	Hypertension	Diabetes
Lithuania	Cognitive impairment	Diabetes	Low self-rated health
Bulgaria	Cognitive impairment	Hypertension	Hypertension
Cyprus	Cognitive impairment	Diabetes	Cognitive impairment
Finland	Cognitive impairment	Hypertension	Diabetes
Latvia	Low self-rated health	Diabetes	Low self-rated health
Malta	Cognitive impairment	Hypertension	Diabetes
Romania	Cognitive impairment	Diabetes	Physical difficulty
Slovakia	Cognitive impairment	Hypertension	Physical difficulty
Indonesia	Cognitive impairment	Cognitive impairment	Hypertension
United States	Physical difficulty	Hypertension	Diabetes
China	Cognitive impairment	Hypertension	Diabetes
Costa Rica	Low self-rated health	Hypertension	Diabetes
Mexico	Low self-rated health	Hypertension	Diabetes
All countries	Cognitive impairment	Hypertension	Diabetes

**Figure 3:**
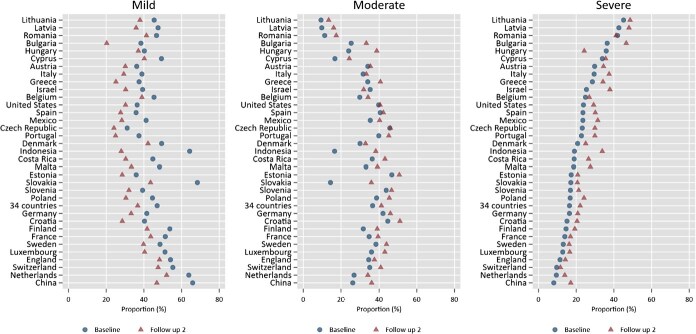
The proportion of older adults who fall into each category at the baseline and follow-up periods in 34 countries. Note: Countries are ordered by the proportion of older adults in severe multimorbidity group at the baseline.

Next, we use class categorization from the previous step to calculate the proportion of older adults falling into each latent category during the baseline and follow-up periods (Figure 3). In the sample of 34 countries (pool dataset), 16% of the older adults are classified as severe, 37% as moderate, and 47% as mild multimorbidity at the baseline (see Table S3 for the complete list). Examining each country’s sample at the baseline, we find that China, The Netherlands, and Switzerland have the lowest proportion of older adults with severe multimorbidity, at 8%, 9%, and 10%, respectively. Conversely, Lithuania, Latvia, and Romania have the highest proportion, with severe multimorbidity prevalence at 45%, 43%, and 42%, respectively.

Except for Hungary and Romania, all countries saw an increase in the proportion of older adults in the severe multimorbidity group during the second follow-up period, which had an average gap of 5 years. Indonesia has the highest increase in this group, by 15 percentage points (a 79% increase compared to the baseline proportion), which can be explained by the 7-year gap in the IFLS survey. For surveys with a 5-year gap, Israel and Bulgaria have the highest increases in the severe multimorbidity group in absolute terms, by 13 and 11 percentage points, respectively (or 52% and 31% relative to the baseline proportion). Meanwhile, in relative terms, besides Indonesia, China, and The Netherlands also have a high increase in the proportion of the severe multimorbidity group by 113% and 56%, respectively. This latter case could be explained by the fact that China and The Netherlands started the lowest proportion of the severe group at the baseline compared to other countries. On the other hand, the proportion of older adults in the moderate multimorbidity group has increased in almost all countries, while the proportion of older adults in the mild multimorbidity group has decreased in all countries.

**Figure 4:**
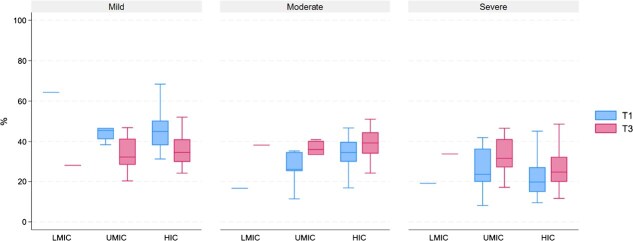
Box plot class proportion by income groups. Note: T1 and T3 are baseline and follow-up 2 periods, respectively. LMIC, UMIC, and HIC stand for lower-middle, upper-middle, and high-income countries, respectively.

**Figure 5:**
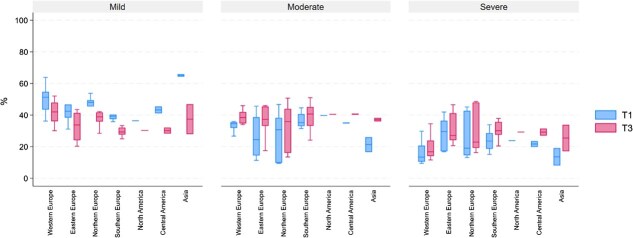
Box plot class proportion by regions. Note: T1 and T3 are baseline and follow-up 2 periods, respectively.

Based on the estimation results in Figure 3, we classified countries by income and region in Figures 4 and 5, respectively. There is substantial variation in the proportion of latent classes of multimorbidity within each group, whether categorized by income or region. Based on country income, we find that on average, the proportion of older adults in severe multimorbidity groups is higher in LMIC and UMICs compared to HIC in the second follow-up period. The condition occurred because of a high rise in the proportion of severe multimorbidity in LMIC and UMICs since the baseline period. Furthermore, LMIC and UMICs have also caught up in the proportion of older adults in the moderate multimorbidity group (Figure 4).

When looking at regions in our sample, we observed a significant increase in the proportion of moderate and severe multimorbidity groups in Asian countries, followed by Central American countries. In the second follow-up period, all regions caught up in the proportion of older adults in the severe multimorbidity group, except for Western Europe (Figure 5).

### Transition probability

**Figure 6:**
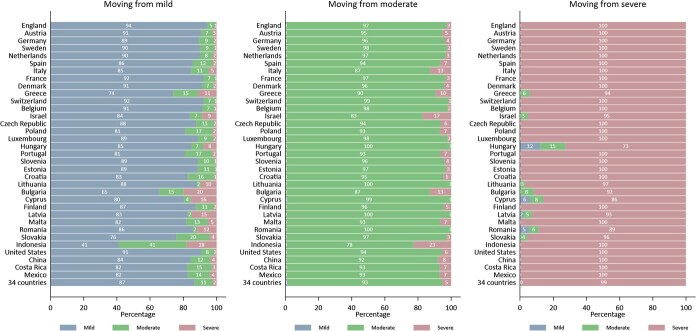
Transition probability for all countries.

Using the pooled dataset, we estimate the transition probability of moving category for an individual from baseline to the first follow-up period as well as from the first follow-up to the second follow-up period as shown in Table 3. Table 3 shows the conditional probability given the initial category (row total). The older adults are more likely to stay in their initial category in the subsequent period indicated by the higher probability in the diagonal cell. The severe category is the most persistent group indicated by almost all individuals staying in the same group over time. Similarly, the mild and moderate multimorbidity groups are also more likely to be persistent. However, the mild and moderate groups tend to move to the severe category in the subsequent period. There is one-fifth of the older adults in the mild category in the initial period move to the moderate category while less than 3% of them move to the severe category. Meanwhile, there are 5% older adults in the moderate group move to the severe group. No individuals moved back from severe or moderate into the mild category from baseline to the first follow-up. However, a small number of people moved from the severe and moderate categories into the mild category from the second follow-up to the first follow-up period (1.8% and 2.8%, respectively). These patterns are driven and observed in the samples from Hungary, Romania, and Cyprus, as indicated in Figure 6. This pattern can be attributed to 2 main reasons. First, these 3 countries experienced a decrease in the proportion of older adults individuals in the severe multimorbidity category. Second, there is a notable decrease in the prevalence of low self-rated health and cognitive impairment in the second follow-up period for these countries, as shown in Table S2 in the Supplementary Materials.

**Table 3:** Transition probability of moving category from initial to subsequent period combining 34 countries.

		**Mild**			
		**Follow up 1**	**Moderate**	**Severe**	**Total**
Baseline	Mild	86.3	11.2	2.5	100
	Moderate	0.0	94.9	5.1	100
	Severe	0.0	0.0	100.0	100
		Follow up 2			
Follow up 1	Mild	86.8	11.3	1.9	100
	Moderate	2.8	91.8	5.4	100
	Severe	1.8	0.0	98.2	100

Figure 6 shows the transition probability for all countries averaging 2 consecutive transitions since the similarity of the transition probability between the first and second periods. The pattern of the multimorbidity transition across countries is quite similar with slightly different magnitudes except for Indonesia (The transition probability for Indonesia indicates a long-term transition over 7 years, while other countries show a medium-term over 2-3 year period.). A high and dominant number indicates the persistence of a multimorbidity status over time. All countries have experienced a transition from mild to moderate and severe multimorbidity category. In a 7-year survey gap, more than half of the older adults in Indonesia move from mild to moderate and severe categories. In the 2- to 3-year survey gap, the percentage of older adults people in the mild category who moved to either moderate or severe categories ranged from 6% in England to 35% in Bulgaria. Additionally, a small percentage of older adults individuals moved from the moderate to severe category, with rates ranging from 1% in Switzerland to 17% in Israel. Most severely multimorbid individuals across the countries remained in the same category over the years except Greece, Israel, Hungary, Lithuania, Bulgaria, Cyprus, Latvia, Romania, and Slovakia. In these 9 countries, the shift from the severe category to a lower level of multimorbidity is driven by the decrease in the prevalence of low self-rated health, as this measure is subjective and reflects perceived health rather than chronic illnesses that persist over time.

### Covariates

Next, we assess the association between covariates and the multimorbidity category using all countries’ samples. Table 4 shows the estimation results (odds ratios) from ordered logit model where the outcome variable multimorbidity is ordinally categorized as follows: 3 for the severe category, 2 for the moderate category, and 1 for the mild category. The older a person is, the more likely he or she is to fall into the moderate and severe categories rather than the mild category. Older adult women are more likely to be in mild or moderate categories, in other words, men tend to have severe multimorbidity than women. Similarly, the older adults with at least secondary school are more likely to be in mild or moderate categories. As a proxy of socioeconomic status, low-education individuals who can be indicated as having a low level of socioeconomic status are associated with severe multimorbidity. Similarly, in transition associations, older individuals, males, and low-education are more likely to progress from mild to more severe multimorbidity categories (Table 5).

**Table 4:** The association between covariates and class of multimorbidity (ordered logit with severe category = 3, moderate category = 2, and mild category = 1, all countries’ samples), odds ratios with 95% CI in the parentheses.

	**Baseline**	**Follow-up 1**	**Follow-up 2**
Age	1.038***	1.035***	1.032***
	(1.037-1.040)	(1.034-1.037)	(1.031-1.033)
Female	1.017	0.990	0.955***
	(0.993-1.041)	(0.968-1.013)	(0.933-0.977)
High education	0.722***	0.716***	0.678***
	(0.702-0.743)	(0.696-0.737)	(0.659-0.697)
Cutoff 1	6.889***	4.461***	3.043***
Cutoff 2	45.406***	30.734***	21.640***
Pseudo R2	0.0538	0.0456	0.0647
Observations	109 037	109 037	109 037
Country dummy	Yes	Yes	Yes

High education is 1 for upper secondary, vocational training, and tertiary, while 0 for less than lower secondary.

^**^ *P* < .05.

^*^ *P* < .1.

^***^ *P* < .001.

**Table 5:** The association between covariates and class transitions (odds ratios with 95% CI in the parentheses).

	Mild	Moderate	Severe
	**Age**		
	Transition from baseline to follow up 1		
Mild	-	1.022 (1.019, 1.026)	1.006 (1.002, 1.011)
Moderate	0.978 (0.975, 0.981)	-	0.984 (0.980, 0.989)
Severe	0.994 (0.989, 0.998)	1.016 (1.012, 1.020)	
	Transition from follow up 1 to follow up 2		
Mild	-	1.021 (1.017, 1.026)	1.005 (0.999, 1.011)
Moderate	0.979 (0.975, 0.983)	-	0.984 (0.979, 0.989)
Severe	0.995 (0.989, 1.001)	1.016 (1.011, 1.021)	
	Female		
	Transition from baseline to follow up 1		
Mild	-	0.992 (0.936, 1.051)	0.936 (0.859, 1.020)
Moderate	1.008 (0.951, 1.068)	-	0.943 (0.872, 1.020)
Severe	1.069 (0.981, 1.165)	1.060 (0.980, 1.147)	
	Transition from follow up 1 to follow up 2		
Mild	-	0.928 (0.866, 0.994)	0.884 (0.798, 0.980)
Moderate	1.078 (1.006, 1.155)	-	0.953 (0.874, 1.039)
Severe	1.131 (1.021, 1.253)	1.049 (0.962, 1.144)	
	High education		
	Transition from baseline to follow up 1		
Mild	-	0.834 (0.774, 0.900)	0.674 (0.600, 0.757)
Moderate	1.199 (1.111, 1.293)	-	0.807 (0.727, 0.896)
Severe	1.485 (1.322, 1.668)	1.239 (1.116, 1.375)	
	Transition from follow up 1 to follow up 2		
Mild	-	0.722 (0.659, 0.791)	0.564 (0.492, 0.648)
Moderate	1.385 (1.264, 1.518)	-	0.782 (0.697, 0.877)
Severe	1.772 (1.543, 2.033)	1.279 (1.140, 1.435)	

High education is 1 for upper secondary, vocational training, and tertiary, while 0 for less than lower secondary. Dashes indicate the reference category. The estimate incorporates country dummies as a control variable.

## Discussions

Our findings are consistent with previous literature that has documented variations of multimorbidity and its rising trends among the older adults in various countries.^45^^,^^46^ However, our analysis highlights the intensity of multimorbidity within the older adult populations that can be classified as an ordinal group (mild, moderate, and severe). The findings reveal that the variation in the intensity of multimorbidity appears to vary widely across countries. For instance, the proportion of older adults in severe multimorbidity ranges from 8% in China to 45% in Lithuania at the baseline period. Over time, the proportion of older adults in severe and moderate multimorbidity tends to increase, with significant variations across countries. Notably, a higher increase in severe multimorbidity is observed in LMIC and UMICs, as well as in regions like Asia and Central America. The study also highlights the persistence of multimorbidity status among the older adults in all countries over time with a tendency to move to the severe category. Similar to previous literature,^15^^,^^34^^,^^37^ this study also finds that diabetes and hypertension are the most common health conditions among the older adults with severe and moderate multimorbidity. Our findings also align with previous literature that identified the risk factors (older, males, and low-education individuals) associated with severe multimorbidity.^15^^,^^34^^,^^37^^,^^47^

Generally, our findings can be interpreted in several ways. First, as the population ages, the increasing severity of multimorbidity in almost all countries serves as a significant alarm for global health, given that prevention and treatment efforts remain inadequate.^48^ Second, multimorbidity imposes a substantial economic burden on healthcare systems due to high direct costs.^49^ For instance, it accounts for 65% of total healthcare expenditures in the United States.^50^ Our findings indicate that LMICs and UMICs tend to have a higher proportion of older adults with severe multimorbidity and are also catching up in moderate multimorbidity compared to HICs. This growing burden could have substantial financial implications for LMICs, which often operate with limited resources and underdeveloped healthcare systems.^51^ Third, most countries exhibit similar morbidity patterns and transitions, suggesting common underlying etiopathogenic factors.^46^ A deeper understanding of these patterns could inform the development of preventive measures to reduce the prevalence of multimorbidity globally. Fourth, the increasing trend of the severity of multimorbidity conditions among the older adults also has significant implications for clinical practice and healthcare quality.^52^ Eventually, the demand for healthcare will grow which will increase the healthcare burden over time. Healthcare systems may require improving services and interventions to meet the needs of severely multimorbid individuals.^53^ A preventive measure of multimorbidity is a key policy to reduce the impact of multimorbidity on the increasing aging population.^54^^,^^55^

This prospective population-based cohort study on multimorbidity has strengths and limitations regarding the data and variables used in the analysis. The strength of this study is the use of longitudinal and comparable-national-representative survey data that covers various countries with different levels of socioeconomic. Although the use of similar questionnaires ensures internal validity, relying on self-reported health indicators might underestimate true prevalence of conditions compared to administrative or clinical databases. This could result in a lower reported prevalence of chronic health conditions like diabetes and hypertension. For instance, Indonesia has a very low prevalence of diabetes, which might be due to low healthcare utilization and access or a lack of awareness among the older adult population. However, a systematic review shows that there was no significant difference in estimated multimorbidity prevalence between self-report and administrative/clinical databases.^56^ We believe that our estimates represent a lower bound of the actual conditions, as the true values could be higher due to underreporting.

## Supplementary Material

Web_Material_kwaf129

## Data Availability

All datasets are available from their respective institutional websites, while the harmonized dataset is provided by the Gateway to Global Aging Data (https://g2aging.org/home). The specific data versions used in this study are listed in the Supplementary Materials Appendix S1.
