# Supplementary material for: Longitudinal pattern of multimorbidity in older adult population: latent transition analysis in 34 countries
Source: Am J Epidemiol. 2025 Jun 13;195(3):653–63. doi: 10.1093/aje/kwaf129 (PMC13016695; doi:10.1093/aje/kwaf129)
Supplement: Web_Material_kwaf129 [file web_material_kwaf129.docx]

***American Journal of Epidemiology* Supplementary Materials**

**Title**: Longitudinal Pattern of Multimorbidity in Older Adult Population: Latent Transition Analysis in 34 Countries

**Authors**: Ridho Al Izzati & Eduwin Pakpahan

List of appendices:

- Appendix S1. List of datasets
- Table S1. List of countries
- Figure S1. BIC
- Figure S2. Item probability for HRS dataset
- Table S2. Prevalence of health status (%), demographics (mean), and sample size by country
- Figure S3. Item probability for each country
- Table S3. The proportion of older adults who fall into each category at the baseline period for all countries (%)

Appendix S1. List of datasets

- CHARLS

This analysis uses data or information from the Harmonized CHARLS dataset and Codebook, Version D as of June 2021 developed by the Gateway to Global Aging Data. The development of the Harmonized CHARLS was funded by the National Institute on Aging (R01 AG030153, RC2 AG036619, R03 AG043052). For more information, please refer to https://g2aging.org/.

This document used CHARLS Waves 1 through 4 as of June 2021. CHARLS is supported by Peking University, the National Natural Science Foundation of China, the National Institute on Aging, and the World Bank.

- CRELES

This analysis uses data or information from the Harmonized CRELES dataset and Codebook, Version A as of August 2016 developed by the Gateway to Global Aging Data. The development of the Harmonized CRELES was funded by the National Institute on Ageing (R01 AG030153, RC2 AG036619, R03 AG043052). For more information, please refer to www.g2aging.org.

This document uses data from the CRELES, released June, 2016. The CRELES Pre-1945 Cohort Study (CRELES Waves 1-3) was conducted by the University of Costa Rica's Centro Centroamericano de Población (CCP) in collaboration with the Instituto de Investigaciones en Salud (INISA), with the support of the Wellcome Trust (grant 072406). The CRELES 1945-1955 Retirement Cohort Study (CRELES Waves 4 and 5) was conducted by the University of Costa Rica’s Centro Centroamericano de Población, in collaboration with the University of California at Berkeley, with funding from the U.S. National Institute on Aging (grant R01AG031716).

- ELSA

This analysis uses data or information from the Harmonized ELSA dataset and Codebook, Version G.3 as of June 2023 developed by the Gateway to Global Aging Data. The development of the Harmonized ELSA was funded by the National Institute on Aging (R01 AG030153, RC2 AG036619, R03 AG043052). For more information, please refer to https://g2aging.org/.

This document uses data from the 38th edition of the ELSA, released April 2023. ELSA is the result of collaboration between University College London (UCL), the Institute for Fiscal Studies (IFS), and NatCen Social Research. Other academic collaborators based at the Universities of Cambridge, Exeter and East Anglia provided expert advice on specific modules. Funding for the first nine waves of ELSA has been provided by the US National Institute on Aging, and a consortium of British Government departments, which are listed below:

• Department of Health;

• Department for Transport;

• Department for Work and Pensions;

• Department for Communities and Local Government (formerly Office of the Deputy Prime Minister);

• HM Treasury;

• Department of Environment, Food and Rural Affairs;

• HM Revenue and Customs (formerly Inland Revenue and HM Customs and Excise);

• Office for National Statistics.

- HRS

Health and Retirement Study, (RAND HRS Longitudinal File 2020 (V1)) public use dataset. Produced and distributed by the University of Michigan with funding from the National Institute on Aging (grant number NIA U01AG009740). Ann Arbor, MI, (March 2023).

RAND HRS Longitudinal File 2020 (V1). Produced by the RAND Center for the Study of Aging, with funding from the National Institute on Aging and the Social Security Administration. Santa Monica, CA (March 2023).

The HRS (Health and Retirement Study) is sponsored by the National Institute on Aging (grant number NIA U01AG009740) and is conducted by the University of Michigan.

- IFLS

IFLS5 was a collaborative effort of RAND and Survey Meter. Funding for IFLS5 was provided by the National Institute on Aging (NIA), grant 2R01 AG026676 05, the National Institute for Child Health and Human Development (NICHD), grant 2R01 HD050764 05A1 and grants from the World Bank, Indonesia and GRM International, Australia from DFAT, the Department of Foreign Affairs and Trade, Government of Australia. http://www.rand.org/FLS/IFLS.

Strauss, J., F. Witoelar, B. Sikoki and A.M. Wattie. “The Fourth Wave of the Indonesia Family Life Survey (IFLS4): Overview and Field Report”. April 2009. WR-675/1-NIA/NICHD.

Strauss, J., F. Witoelar, and B. Sikoki. “The Fifth Wave of the Indonesia Family Life Survey (IFLS5): Overview and Field Report”. March 2016. WR-1143/1-NIA/NICHD.

- MHAS

Michaels-Obregon, Alejandra, Drystan Phillips, Jenny Wilkens, Rebeca Wong, and Jinkook Lee. “Harmonized MHAS, Version C.2.” Gateway to Global Aging Data, 2023. https://doi.org/10.34729/06K4-ZG60.

This analysis uses data or information from the Harmonized MHAS dataset and Codebook, Version C.2 as of August 2023 developed by the Gateway to Global Aging Data in collaboration with the MHAS research team. The development of the Harmonized MHAS was funded by the National Institute on Aging (R01 AG030153). The Harmonized MHAS data files and documentation are public use and available at www.MHASweb.org. The MHAS (Mexican Health and Aging Study) receives support from the National Institutes of Health/National Institute on Aging (R01 AG018016) in the United States and the Instituto Nacional de Estadística y Geografía (INEGI) in Mexico. For more information about the Harmonization project, please refer to www.g2aging.org.”

This document uses data from the MHAS datasets as of July 2020. The MHAS (Mexican Health and Aging Study) is partly sponsored by the National Institutes of Health/National Institute on Aging (grant number NIH R01AG018016) in the United States and the Instituto Nacional de Estadística y Geografía (INEGI) in Mexico. Data files and documentation are public use and available at [www.MHASweb.org](http://www.MHASweb.org).

- SHARE

This analysis uses data or information from the Harmonized SHARE dataset and Codebook, Version F as of June 2022 developed by the Gateway to Global Aging Data. The development of the Harmonized SHARE was funded by the National Institute on Aging (R01 AG030153, RC2 AG036619, R03 AG043052). For moreinformation, please refer to “https://g2aging.org/”.

This document uses data from SHARE Waves 1, 2, 3, 4, 5, 6, 7 and 8 release 8.0.0 as of February 2022.. The SHARE data collection has been funded by the European Commission through the 5th framework programme (project QLK6-CT-2001-00360 in the thematic programme Quality of Life). Further support by the European Commission through the 6th framework programme (projects SHARE-I3, RII-CT-2006-062193, as an Integrated Infrastructure Initiative, COMPARE, CIT5-CT-2005-028857, as a project in Priority 7, Citizens and Governance in a Knowledge Based Society, and SHARE-LIFE (CIT4-CT-2006-028812)) and through the 7th framework programme (SHARE-PREP (No 211909), SHARE-LEAP (No 227822) and M4 (No 261982)) is gratefully acknowledged. Substantial co-funding for add-ons such as the intensive training programme for SHARE interviewers came from the US National Institute on Aging (U01 AG09740-13S2, P01 AG005842, P01 AG08291, P30 AG12815, R21 AG025169, Y1-AG-4553-01, IAG BSR06-11 and OGHA 04-064). Substantial funding for the central coordination of SHARE came from the German Federal Ministry for Education and Research (Bundesministerium für Bildung und Forschung, BMBF). See http://www.share-project.org/contact-organisation/funding.html for a full list of funding institutions.

Table S1. List of countries

| Country | Baseline | Follow up 1 | Follow up 2 | Income group | Region |
| --- | --- | --- | --- | --- | --- |
| England | 2014/2015 (Wave 7) | 2016/2017 (Wave 8) | 2018/2019 (Wave 9) | HIC | Western Europe |
| Austria | 2017 (Wave 7) | 2019/2020 (Wave 8) | 2021/2022 (Wave 9) | HIC | Western Europe |
| Germany | 2017 (Wave 7) | 2019/2020 (Wave 8) | 2021/2022 (Wave 9) | HIC | Western Europe |
| Sweden | 2017 (Wave 7) | 2019/2020 (Wave 8) | 2021/2022 (Wave 9) | HIC | Northern Europe |
| Netherlands | 2013 (Wave 5) | 2019/2020 (Wave 8) | 2021/2022 (Wave 9) | HIC | Western Europe |
| Spain | 2017 (Wave 7) | 2019/2020 (Wave 8) | 2021/2022 (Wave 9) | HIC | Southern Europe |
| Italy | 2017 (Wave 7) | 2019/2020 (Wave 8) | 2021/2022 (Wave 9) | HIC | Southern Europe |
| France | 2017 (Wave 7) | 2019/2020 (Wave 8) | 2021/2022 (Wave 9) | HIC | Western Europe |
| Denmark | 2017 (Wave 7) | 2019/2020 (Wave 8) | 2021/2022 (Wave 9) | HIC | Northern Europe |
| Greece | 2017 (Wave 7) | 2019/2020 (Wave 8) | 2021/2022 (Wave 9) | HIC | Southern Europe |
| Switzerland | 2017 (Wave 7) | 2019/2020 (Wave 8) | 2021/2022 (Wave 9) | HIC | Western Europe |
| Belgium | 2017 (Wave 7) | 2019/2020 (Wave 8) | 2021/2022 (Wave 9) | HIC | Western Europe |
| Israel | 2017 (Wave 7) | 2019/2020 (Wave 8) | 2021/2022 (Wave 9) | HIC | Southern Europe |
| Czech Republic | 2017 (Wave 7) | 2019/2020 (Wave 8) | 2021/2022 (Wave 9) | HIC | Eastern Europe |
| Poland | 2017 (Wave 7) | 2019/2020 (Wave 8) | 2021/2022 (Wave 9) | HIC | Eastern Europe |
| Luxembourg | 2017 (Wave 7) | 2019/2020 (Wave 8) | 2021/2022 (Wave 9) | HIC | Western Europe |
| Hungary | 2017 (Wave 7) | 2019/2020 (Wave 8) | 2021/2022 (Wave 9) | HIC | Eastern Europe |
| Portugal | 2015 (Wave 6) | 2017/2018 (Wave 7) | 2021/2022 (Wave 9) | HIC | Southern Europe |
| Slovenia | 2017 (Wave 7) | 2019/2020 (Wave 8) | 2021/2022 (Wave 9) | HIC | Southern Europe |
| Estonia | 2017 (Wave 7) | 2019/2020 (Wave 8) | 2021/2022 (Wave 9) | HIC | Northern Europe |
| Croatia | 2017 (Wave 7) | 2019/2020 (Wave 8) | 2021/2022 (Wave 9) | HIC | Southern Europe |
| Lithuania | 2017 (Wave 7) | 2019/2020 (Wave 8) | 2021/2022 (Wave 9) | HIC | Northern Europe |
| Bulgaria | 2017 (Wave 7) | 2019/2020 (Wave 8) | 2021/2022 (Wave 9) | UMIC | Eastern Europe |
| Cyprus | 2017 (Wave 7) | 2019/2020 (Wave 8) | 2021/2022 (Wave 9) | HIC | Southern Europe |
| Finland | 2017 (Wave 7) | 2019/2020 (Wave 8) | 2021/2022 (Wave 9) | HIC | Northern Europe |
| Latvia | 2017 (Wave 7) | 2019/2020 (Wave 8) | 2021/2022 (Wave 9) | HIC | Northern Europe |
| Malta | 2017 (Wave 7) | 2019/2020 (Wave 8) | 2021/2022 (Wave 9) | HIC | Southern Europe |
| Romania | 2017 (Wave 7) | 2019/2020 (Wave 8) | 2021/2022 (Wave 9) | UMIC | Eastern Europe |
| Slovakia | 2017 (Wave 7) | 2019/2020 (Wave 8) | 2021/2022 (Wave 9) | HIC | Eastern Europe |
| Indonesia | 2007/2008 (Wave 4) | - | 2014/2015 (Wave 5) | LMIC | Asia |
| United States | 2012 (Wave 11) | 2014 (Wave 12) | 2016 (Wave 13) | HIC | North America |
| China | 2013 (Wave 2) | 2015 (Wave 3) | 2018 (Wave 4) | UMIC | Asia |
| Costa Rica | 2005 (Wave 1) | 2007 (Wave 2) | 2009 (Wave 3) | UMIC | Central America |
| Mexico | 2012 (Wave 3) | 2015 (Wave 4) | 2018 (Wave 5) | UMIC | Central America |

Note: HIC=High-income country, UMIC=upper-middle-income country, LMIC=lower-middle-income country. Region of the country is based on Standard Country or Area Codes for Statistical Use by the Statistics Division of the United Nations (https://unstats.un.org/unsd/methodology/m49/).

Figure S1. BIC


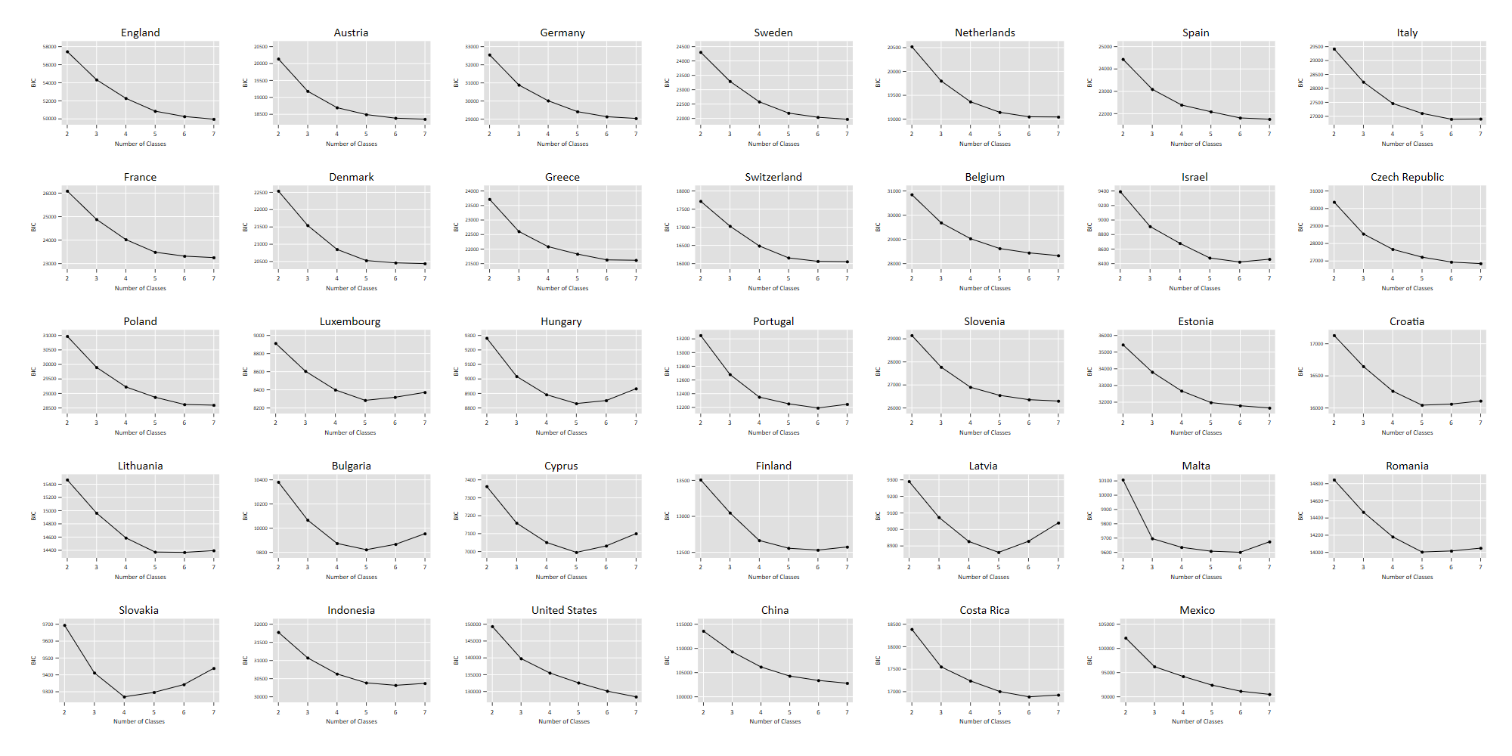


Figure S2. Item probability for HRS dataset


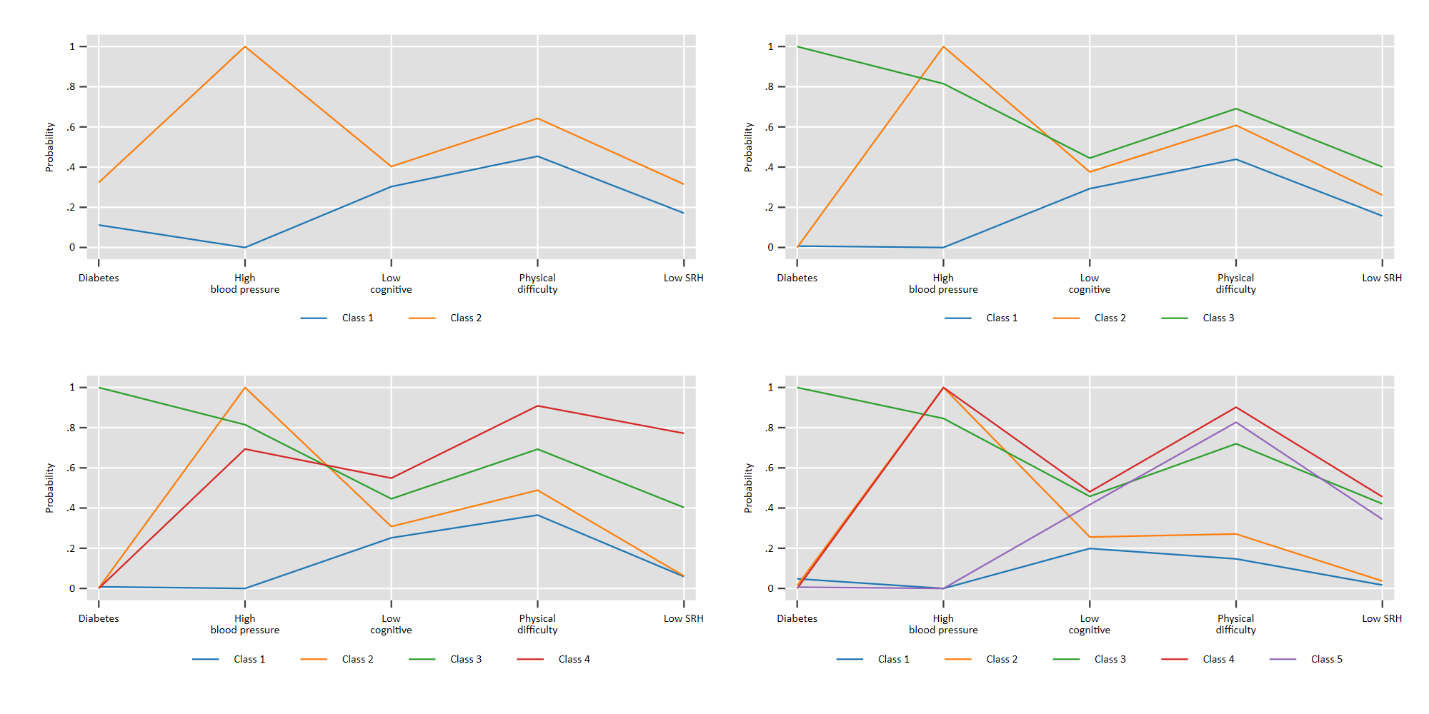


Table S2. Prevalence of health status (%), demographics (mean), and sample size by country

| Country | Diabetes | | | Hypertension | | | Low cognitive | | | Physical difficulty | | | Low self-rated health | | | Age | Female | High school | Sample size |
| --- | --- | --- | --- | --- | --- | --- | --- | --- | --- | --- | --- | --- | --- | --- | --- | --- | --- | --- | --- |
|  | *1* | *2* | *3* | *1* | *2* | *3* | *1* | *2* | *3* | *1* | *2* | *3* | *1* | *2* | *3* |  |  |  |  |
| England | 11 | 13 | 14 | 42 | 45 | 47 | 28 | 30 | 31 | 45 | 48 | 48 | 25 | 27 | 27 | 67 | 0.55 | 0.74 | 7132 |
| Austria | 17 | 18 | 20 | 60 | 64 | 67 | 33 | 34 | 37 | 43 | 47 | 46 | 34 | 35 | 36 | 70 | 0.61 | 0.77 | 2244 |
| Germany | 16 | 19 | 21 | 55 | 60 | 63 | 33 | 37 | 39 | 42 | 48 | 50 | 40 | 40 | 41 | 67 | 0.53 | 0.90 | 3007 |
| Sweden | 13 | 15 | 16 | 49 | 53 | 56 | 37 | 39 | 39 | 34 | 42 | 43 | 27 | 28 | 30 | 71 | 0.54 | 0.69 | 2410 |
| Netherlands | 9 | 13 | 13 | 32 | 40 | 43 | 31 | 43 | 39 | 28 | 38 | 36 | 22 | 29 | 30 | 64 | 0.55 | 0.60 | 2237 |
| Spain | 25 | 27 | 33 | 61 | 66 | 73 | 70 | 69 | 70 | 40 | 49 | 50 | 43 | 45 | 49 | 71 | 0.56 | 0.22 | 2366 |
| Italy | 17 | 21 | 22 | 58 | 67 | 69 | 61 | 65 | 58 | 36 | 45 | 44 | 42 | 46 | 45 | 68 | 0.56 | 0.32 | 3330 |
| France | 14 | 16 | 16 | 44 | 49 | 52 | 40 | 42 | 40 | 40 | 47 | 45 | 34 | 36 | 35 | 68 | 0.58 | 0.65 | 2450 |
| Denmark | 9 | 10 | 10 | 43 | 48 | 51 | 31 | 33 | 33 | 29 | 33 | 35 | 24 | 24 | 26 | 66 | 0.54 | 0.85 | 2510 |
| Greece | 16 | 20 | 26 | 57 | 65 | 72 | 57 | 59 | 54 | 45 | 47 | 53 | 30 | 36 | 34 | 68 | 0.57 | 0.50 | 2392 |
| Switzerland | 10 | 11 | 12 | 42 | 47 | 49 | 31 | 33 | 37 | 29 | 35 | 35 | 19 | 20 | 21 | 69 | 0.55 | 0.81 | 1874 |
| Belgium | 14 | 17 | 18 | 46 | 52 | 54 | 36 | 39 | 38 | 43 | 50 | 49 | 28 | 30 | 30 | 67 | 0.56 | 0.66 | 3574 |
| Israel | 34 | 40 | 40 | 58 | 62 | 67 | 46 | 51 | 54 | 32 | 44 | 43 | 40 | 46 | 45 | 70 | 0.59 | 0.67 | 867 |
| Czech Republic | 23 | 27 | 28 | 66 | 70 | 72 | 42 | 40 | 39 | 45 | 48 | 48 | 27 | 28 | 28 | 69 | 0.62 | 0.64 | 2906 |
| Poland | 17 | 24 | 24 | 52 | 62 | 67 | 60 | 65 | 64 | 49 | 55 | 47 | 48 | 52 | 48 | 65 | 0.56 | 0.72 | 3356 |
| Luxembourg | 13 | 15 | 16 | 44 | 52 | 54 | 32 | 34 | 29 | 41 | 47 | 44 | 33 | 33 | 31 | 66 | 0.56 | 0.63 | 840 |
| Hungary | 24 | 31 | 38 | 69 | 79 | 84 | 49 | 43 | 34 | 45 | 48 | 49 | 54 | 46 | 42 | 68 | 0.61 | 0.75 | 911 |
| Portugal | 23 | 27 | 30 | 58 | 66 | 72 | 66 | 74 | 71 | 55 | 52 | 58 | 68 | 68 | 71 | 66 | 0.57 | 0.20 | 1203 |
| Slovenia | 17 | 21 | 21 | 57 | 64 | 65 | 51 | 48 | 55 | 41 | 45 | 47 | 38 | 36 | 37 | 68 | 0.60 | 0.68 | 2924 |
| Estonia | 18 | 20 | 21 | 62 | 67 | 69 | 47 | 42 | 45 | 51 | 53 | 55 | 72 | 72 | 71 | 69 | 0.65 | 0.78 | 3731 |
| Croatia | 15 | 18 | 21 | 56 | 65 | 69 | 54 | 52 | 58 | 53 | 55 | 54 | 45 | 48 | 40 | 65 | 0.57 | 0.41 | 1808 |
| Lithuania | 9 | 12 | 13 | 53 | 64 | 71 | 59 | 66 | 60 | 47 | 49 | 51 | 60 | 59 | 60 | 66 | 0.64 | 0.81 | 1508 |
| Bulgaria | 12 | 18 | 20 | 55 | 70 | 76 | 62 | 51 | 58 | 43 | 49 | 48 | 41 | 43 | 45 | 66 | 0.59 | 0.66 | 1021 |
| Cyprus | 17 | 26 | 25 | 46 | 59 | 62 | 66 | 70 | 61 | 34 | 53 | 38 | 34 | 43 | 38 | 69 | 0.61 | 0.45 | 808 |
| Finland | 15 | 17 | 18 | 42 | 50 | 54 | 41 | 39 | 39 | 30 | 35 | 40 | 39 | 40 | 39 | 66 | 0.54 | 0.72 | 1348 |
| Latvia | 10 | 13 | 16 | 45 | 63 | 71 | 49 | 54 | 45 | 42 | 49 | 44 | 65 | 76 | 70 | 66 | 0.65 | 0.83 | 978 |
| Malta | 19 | 24 | 26 | 45 | 54 | 60 | 57 | 62 | 55 | 34 | 24 | 39 | 40 | 43 | 46 | 66 | 0.56 | 0.51 | 975 |
| Romania | 11 | 14 | 18 | 46 | 58 | 67 | 63 | 59 | 56 | 45 | 48 | 44 | 49 | 49 | 46 | 65 | 0.58 | 0.50 | 1534 |
| Slovakia | 7 | 12 | 18 | 24 | 40 | 52 | 53 | 47 | 55 | 30 | 36 | 37 | 21 | 22 | 20 | 62 | 0.54 | 0.89 | 1073 |
| Indonesia | 3 |  | 8 | 19 |  | 34 | 76 |  | 80 | 17 |  | 43 | 19 |  | 35 | 58 | 0.52 | 0.17 | 3429 |
| United States | 24 | 26 | 29 | 59 | 62 | 65 | 39 | 40 | 42 | 57 | 60 | 61 | 27 | 30 | 30 | 67 | 0.59 | 0.82 | 16823 |
| China | 8 | 11 | 17 | 30 | 37 | 47 | 71 | 76 | 83 | 50 | 52 | 57 | 25 | 25 | 30 | 62 | 0.51 | 0.11 | 11557 |
| Costa Rica | 19 | 23 | 27 | 50 | 58 | 66 | 34 | 38 | 32 | 43 | 52 | 59 | 49 | 45 | 46 | 78 | 0.55 | 0.08 | 1712 |
| Mexico | 24 | 28 | 31 | 52 | 61 | 66 | 29 | 33 | 32 | 52 | 58 | 58 | 64 | 69 | 67 | 64 | 0.58 | 0.13 | 12199 |
| All countries | 16 | 20 | 22 | 49 | 56 | 60 | 46 | 46 | 48 | 45 | 51 | 51 | 37 | 39 | 40 | 66 | 0.57 | 0.54 | 109037 |

Note: 1=Baseline, 2=Follow-up 2, 3=Follow-up 2. Column Age, Women, and High school show the mean of the sample.

Figure S3. Item probability for each country


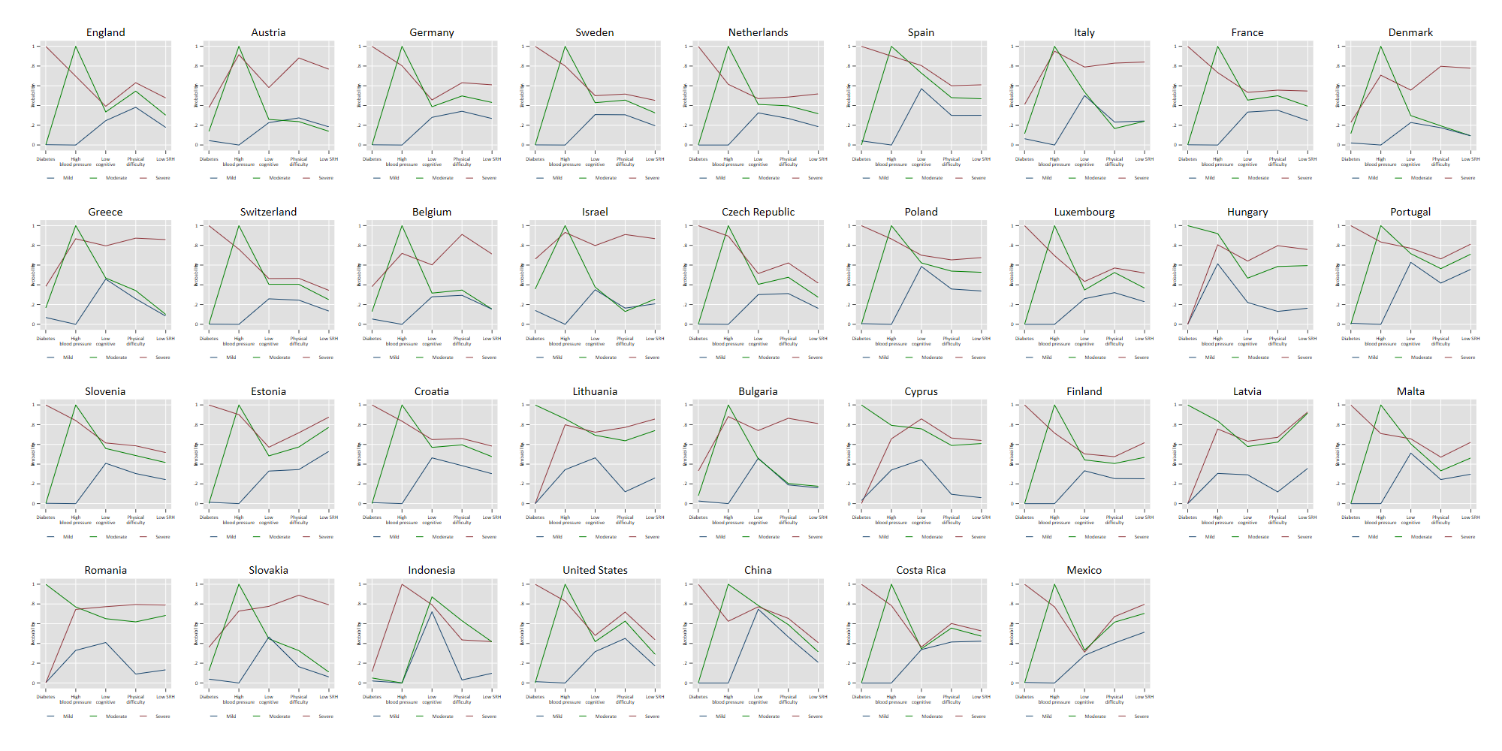


Table S3. The proportion of older adults who fall into each category at the baseline period for all countries (%)

| Country | Mild | | | Moderate | | | Severe | | | Entropy |
| --- | --- | --- | --- | --- | --- | --- | --- | --- | --- | --- |
|  | Baseline | FU1 | FU2 | Baseline | FU1 | FU2 | Baseline | FU1 | FU2 |  |
| England | 54 | 51 | 48 | 34 | 36 | 37 | 11 | 13 | 14 | 0.991 |
| Austria | 36 | 34 | 30 | 34 | 34 | 35 | 30 | 32 | 34 | 0.927 |
| Germany | 42 | 37 | 33 | 42 | 44 | 46 | 16 | 19 | 21 | 0.986 |
| Sweden | 49 | 44 | 40 | 38 | 42 | 44 | 13 | 15 | 16 | 0.986 |
| Netherlands | 64 | 56 | 52 | 27 | 32 | 34 | 9 | 12 | 14 | 0.981 |
| Spain | 36 | 32 | 28 | 41 | 42 | 42 | 24 | 26 | 30 | 0.954 |
| Italy | 39 | 34 | 29 | 32 | 33 | 33 | 30 | 33 | 37 | 0.911 |
| France | 51 | 47 | 44 | 35 | 37 | 39 | 14 | 16 | 17 | 0.986 |
| Denmark | 49 | 47 | 42 | 30 | 31 | 33 | 21 | 22 | 25 | 0.936 |
| Greece | 37 | 31 | 25 | 34 | 37 | 41 | 29 | 32 | 34 | 0.928 |
| Switzerland | 55 | 51 | 48 | 35 | 38 | 41 | 10 | 11 | 12 | 0.989 |
| Belgium | 45 | 43 | 39 | 30 | 32 | 34 | 25 | 25 | 27 | 0.943 |
| Israel | 39 | 36 | 30 | 35 | 29 | 32 | 25 | 35 | 38 | 0.894 |
| Czech Republic | 31 | 28 | 24 | 46 | 46 | 46 | 23 | 26 | 30 | 0.979 |
| Poland | 45 | 36 | 30 | 39 | 43 | 45 | 17 | 21 | 24 | 0.974 |
| Luxembourg | 51 | 45 | 40 | 36 | 40 | 43 | 13 | 15 | 17 | 0.982 |
| Hungary | 40 | 39 | 37 | 24 | 31 | 39 | 36 | 30 | 24 | 0.873 |
| Portugal | 37 | 29 | 25 | 40 | 44 | 45 | 23 | 27 | 30 | 0.978 |
| Slovenia | 39 | 35 | 32 | 44 | 46 | 47 | 17 | 20 | 21 | 0.989 |
| Estonia | 36 | 32 | 28 | 47 | 49 | 51 | 17 | 19 | 21 | 0.986 |
| Croatia | 40 | 33 | 29 | 45 | 49 | 51 | 15 | 18 | 20 | 0.98 |
| Lithuania | 45 | 39 | 38 | 9 | 12 | 13 | 45 | 49 | 49 | 0.933 |
| Bulgaria | 38 | 28 | 20 | 25 | 32 | 33 | 36 | 40 | 47 | 0.875 |
| Cyprus | 49 | 39 | 40 | 17 | 22 | 24 | 34 | 39 | 36 | 0.909 |
| Finland | 54 | 46 | 42 | 32 | 37 | 39 | 15 | 17 | 19 | 0.979 |
| Latvia | 48 | 36 | 36 | 10 | 13 | 16 | 43 | 52 | 48 | 0.891 |
| Malta | 48 | 40 | 33 | 33 | 37 | 39 | 19 | 23 | 28 | 0.979 |
| Romania | 47 | 43 | 41 | 11 | 15 | 18 | 42 | 42 | 41 | 0.915 |
| Slovakia | 68 | 56 | 44 | 14 | 25 | 36 | 17 | 20 | 21 | 0.947 |
| Indonesia | 64 |  | 28 | 17 |  | 38 | 19 |  | 34 | 0.814 |
| United States | 36 | 33 | 30 | 40 | 40 | 40 | 24 | 26 | 29 | 0.989 |
| China | 66 | 59 | 47 | 26 | 30 | 36 | 8 | 11 | 17 | 0.982 |
| Costa Rica | 45 | 37 | 30 | 36 | 40 | 43 | 19 | 23 | 27 | 0.999 |
| Mexico | 41 | 33 | 28 | 35 | 39 | 40 | 24 | 28 | 31 | 0.984 |
| All countries | 47 | 41 | 37 | 37 | 40 | 41 | 16 | 19 | 22 | 0.980 |

Note: FU1=Follow-up 1, FU2=Follow-up 2.
